# Testing the influence of harm reduction messages on health risk attitudes, injunctive norms and perceived behavioral control

**DOI:** 10.1186/s12954-023-00846-2

**Published:** 2023-08-18

**Authors:** Sherri Jean Katz, Elisia Cohen, Dorothy Hatsukami

**Affiliations:** 1 Hubbard School of Journalism and Mass Communication, University of Minnesota, Minneapolis, USA; 2 Department of Psychiatry and Behavioral Sciences, University of Minnesota, Minneapolis, USA

**Keywords:** E-cigarettes, Harm reduction messages, Smoking cues

## Abstract

**Background:**

E-cigarettes can potentially be a harm reduction pathway for adults who smoke and who are seeking to make the complete switch from cigarettes. However, often people who smoke believe that e-cigarettes are just as damaging as cigarettes to their health. From a harm reduction perspective, the key question is whether providing information about the reduced toxicant intake of e-cigarettes, compared to cigarettes, could influence their perceptions and whether there are certain message features that might further support this transition.

**Methods:**

In this experiment (*n* = 305), we test whether a harm reduction (reduced toxicant intake, complete switch) message will influence the health risk attitudes, injunctive norms and perceived behavioral control of people who smoke, compared to those who do not view a message and whether including a “smoking cue” within the message influences their response.

**Results:**

Results indicate that those who viewed the harm reduction message with a smoking cue had lower health risk attitudes than those who did not view a message (*p* = 0.025) and higher injunctive norms than those who viewed the message without a smoking cue (*p* = 0.006).

**Conclusions:**

These findings demonstrate that a harm reduction message with a smoking cue can influence the perceptions of adults who smoke, lowering health risk attitudes and increasing injunctive norms.

## Introduction

E-cigarettes have been called a reduced harm product that can potentially ease the public health burden of tobacco if people who smoke transition completely [1, 2]. One challenge to complete transition is that prior research has highlighted that many adults who smoke believe e-cigarettes are as harmful as combustible cigarettes [3]. For example, in a study of 1843 US adults who smoke, 53% of those who were not using e-cigarettes, highlighted a concern over the safety of the product as a reason not to use them, while 52% of them noted they were skeptical that e-cigarettes could help them completely quit smoking [4]. This sentiment was also relevant in a focus group study distinguishing perceptions of *nicotine* and *addiction* among US adults, conducted in Spring 2020 that included people who smoked, who smoked and vaped, who used to smoke, and who did not smoke or vape [5]. Participants perceived that it was nicotine, rather than smoking, that led to disease, with perceptions influenced by how individuals viewed the concept of addiction and whether they smoked or vaped [5]. Interestingly, addiction was viewed by participants who smoked cigarettes as not only about a chemical response to nicotine, but rather about the behavioral aspects of smoking, which is noteworthy because e-cigarette use mimics many of these behavioral factors [5].

The key question in this current study is whether informing people who smoke cigarettes about the reduced toxicant intake of e-cigarettes, compared to cigarettes, within a testimonial message about a person who has made the complete switch to vaping, could influence these perceptions. In other words, can a message designed to address both the safety concerns and the efficacy skepticism change how adults who smoke perceive e-cigarettes and what message features might further support this transition.

### E-cigarettes and smoking cessation

A recent network meta-analysis has highlighted the potential of e-cigarettes as a reduced harm product in support of smoking cessation, noting that participants assigned to use nicotine e-cigarettes had greater rates of smoking abstinence than those assigned to use a licensed nicotine replacement therapy (NRT) or those assigned to a non-nicotine control condition [6]. Indeed, of the many reduced harm product options, e-cigarettes provide a behavioral experience that most closely mimics cigarette smoking, and prior research mentioned above has shown that people who smoke view the behavioral aspects of smoking as a part of the addiction [5]. One concern is that there are high rates of people who both smoke and vape among those who begin using them [7], and research has highlighted that these individuals are a multifaceted group with specific factors predicting whether or not a person makes the complete switch away from smoking cigarettes and therein whether or not e-cigarettes are actually harm reducing for that individual [8]. However, in recognition that there is at least the potential for e-cigarettes to be used as a harm reduction pathway for some adults who smoke cigarettes [9], it is important to identify what are the characteristics of a health message that best facilitates persuading people who smoke cigarettes to make a complete switch from cigarettes.

### Smoking cues

When considering how best to inform adults who smoke that using e-cigarettes leads to lower exposure of harmful chemicals compared to cigarettes, it is important to consider both the arguments in the message (what they are told) and the format of the message (how it looks). Indeed, research on the heuristic–systematic model has highlighted that the arguments in the message are processed logically (systematically) with a focus on the strength of the message, while key imagery, can be processed simultaneously through a heuristic pathway, and that both of these dual processes support the persuasiveness of the message [10]. Therefore, it is important to acknowledge not only the key arguments of the message (i.e., e-cigarettes are less toxic than cigarettes), but also what the message looks like (i.e., whether or not there are certain visual cues on the message, such as a cloud of smoke) when testing its effectiveness.

When thinking about smoking cessation, one visual imagery characteristic to consider is the influence of a smoking cue, which refers to visual imagery related to smoking use, such as a cloud of smoke [11]. Research has demonstrated that including smoking cues in public service announcements weakens the strength of the argument of the message [12] and lowers self-efficacy to refrain from smoking [13]. Therein, it may be tempting to simply conclude that smoking cues should not be used in a message that is designed to persuade people who smoke to make the complete switch to e-cigarettes. However, there is another, more complicated rationale to consider. Because smoking cues remind people who smoke of their cigarettes [11], it is possible that including a smoking cue on the reduced harm message about e-cigarettes might help connect the use of e-cigarettes to the experience of smoking cigarettes. In other words, including the smoking cue might help the person who smokes cigarettes to perceive e-cigarettes as a replacement for cigarettes, potentially increasing the persuasiveness of the reduced harm message.

It is important to note that while this study was conceptualized as including a smoking cue, a distinction has since been made in the literature between smoking cues and vaping cues, wherein the visual presentation of the specific product is what distinguishes between the two [14], and the manipulation used in our study, wherein no specific product is shown in relation to the smoke, is what has been recently called a “neutral cue” [15]. The findings in this recent literature on whether or not cues influence smoking or vaping urges is mixed, with findings that show that vaping cues in e-cigarette advertisements increase the urge of those who smoke everyday to smoke a cigarette [14], and alternate findings that show that smoking, vaping, and neutral cues in e-cigarette public service announcements (PSAs) do not influence smoking desire [15]. This latter study did find that vaping cues, in relation to neutral ones, did seem to increase vaping desire [15]. While we refer to the visual cue used in this current study as a “smoking cue,” as it was initially conceptualized, it is important to acknowledge that it might be more accurately described as a “neutral cue,” and it might connect the person who smokes to their vaping behavior, particularly if they both smoke and vape. Regardless, it is important to test the influence of this cue on the effectiveness of a reduced harm message, which might be perceived differently than the e-cigarette advertisements or vaping PSAs used in the cited studies [14, 15].

### Theoretical rationale and hypotheses

From a reasoned action perspective, the intention to engage in a particular behavior, such as making the complete switch from cigarettes, is predicted, at least in part, by attitudes, perceived norms, and perceived behavioral control [16]. Media messages may influence these attitudinal, normative, and perceived behavioral control constructs, and in the case of a reduced harm message would be expected to predict intentions to make the switch from cigarette smoking to e-cigarettes. In recent studies, normative information in media messages has been shown to influence normative perceptions [17, 18]. For example, the presentation of “individual use depictions,” or portrayals of product use by a specific person, influences normative perceptions, including injunctive norms [18]. The purpose of this study is to determine whether adults who regularly smoke cigarettes and view a reduced toxicant intake (reduced harm/complete switch) message that portrays an individual making the complete switch report (H1) lower health risk attitudes, (H2) higher injunctive norms, and (H3) higher perceived behavioral control than participants who do not view a message. Additionally, drawing upon the theoretical concept of a “smoking cue” [12, 13], we might ask whether a message with or without a “smoking cue” is associated with (RQ1) lower health risk attitudes, (RQ2) higher injunctive norms, and (RQ3) higher perceived behavioral control?

## Material and methods

### Participants

An online experiment was conducted using a panel of pre-registered participants who smoke cigarettes from Amazon Mechanical Turk (mTurk). The data were submitted to OSF, and the link to the public data set is https://osf.io/VB4KD. The final sample consisted of 305 adults who smoke cigarettes after 10 participants were removed from analysis for not completing the study within the designated timeframe, one participant was removed for failing an attention check question, and one participant was removed for no longer smoking cigarettes. They ranged in age from 21 to 76 (*M* = 39.39. SD = 11.22). In response to a question asking how they *define their sexual identity*, 134 participants selected *male*; 170 selected *female*, and one participant indicated another category *not listed*. The participants were primarily white (*n* = 260), with 21 participants stating they were Black/African American, 7 stating they were Asian, 4 stating they were American Indian/Alaskan Native, 1 stating they were Native Hawaiian or Pacific Islander, and 12 selecting more than one category. Additionally, 30 participants noted they were Hispanic. Participants were English-speaking, with 303 confirming they had spoken the language for over 10 years.

The majority of participants (*n* = 295) smoked cigarettes *everyday*, with just a few (*n* = 10) indicating they smoked *some days*. Almost all of the participants had tried an e-cigarette in the past (e-cigarette ever-use, *n* = 271), and almost half had used them in the past 30 days (current e-cigarette use, *n* = 140). Additionally, most participants recalled seeing an advertisement for e-cigarettes (*n* = 233), describing in an open-ended question that they had seen them across a vast variety of settings and media. The majority of participants had not stopped smoking cigarettes for 1 day or longer in an effort to quit, while 131 participants had stopped for 1 day or longer, and 96 participants had two or more quit attempts.

### Procedure

First, participants agreed to an IRB approved consent form. Next, they responded to demographic and tobacco use questions. Participants were randomly assigned to view one of three message conditions: reduced harm message with smoking cue/reduced harm message without smoking cue/and control no message condition. Finally, participants responded to dependent measures and were debriefed.

#### Stimulus materials

**Fig. 1:**
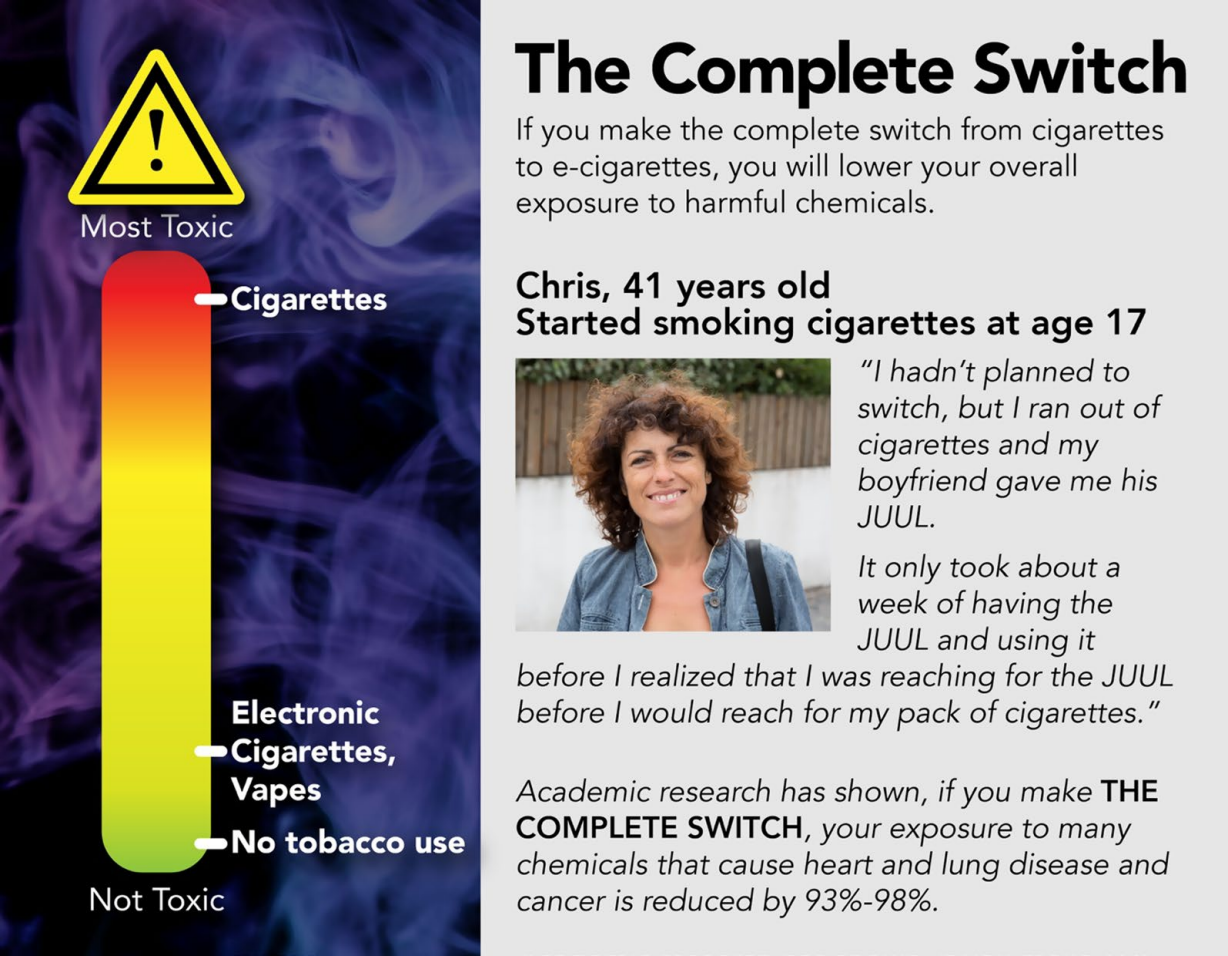
Example stimuli—female version, with smoking cue. *Notes*: The smoking cue is the image of smoke behind the toxicant indicator. The version without a smoking cue had a black background (no smoke) behind the toxicant indicator. Photographs used in testimonials were purchased through a stock image service, and the “testimonials” were created for the study. The story, names, characters, and incidents portrayed in the testimonials are fictitious, and no identification with actual persons should be inferred

The reduced harm stimuli were developed through an iterative design process (Fig. 1). The message featured: (1) a toxic-indicator, showing cigarettes in red at the top, no tobacco use in green at the bottom, and e-cigarettes in yellow–green toward the bottom; (2) a reduced exposure statement; and (3) a testimonial and photograph of a person who had made the complete switch from smoking cigarettes to vaping (the person was gender-matched to the participant). The smoking cue referred to the misty vapor behind the “toxic-indicator.” The version without the smoking cue just had a black background behind the “toxic-indicator.”

### Measures

#### Health risk attitudes

The health risk attitude scale was developed by averaging the responses to two questions based on attitudinal beliefs about e-cigarettes that were identified in prior research [19]. *How risky are electronic cigarettes (e-cigarettes/vapes/JUULs)?* And, *compared to traditional cigarettes, is using electronic cigarettes (e-cigarettes/vapes/JUULs) everyday risky for one’s health?* (1) *not risky at all* to (4) *very risky* (*N* = 305, *M* = 2.35, SD = 0.72, *r* = 0.79. A confirmatory factor analysis shows that both items load on one component.

#### Injunctive norms

The injunctive norm scale was developed by averaging the responses to two questions, adapted from prior research using the reasoned action approach to fit the “complete switch” message topic [20]: *How do you think most people important to you would feel about you using e-cigarettes (e-cigarettes/vapes/JUULs) everyday? They would…* and *How do you think most people important to you would feel about you completely switching to e-cigarettes (e-cigarettes/vapes/JUULs) from traditional cigarettes everyday? They would…* Response options included (1) *strongly disapprove* to (5) *strongly approve* (*N* = 305, *M* = 3.36, SD = 0.85, *r* = 0.59). A confirmatory factor analysis shows that both items load on one component.

#### Perceived behavioral control

To measure perceived behavioral control, participants responded to the prompt: *My completely switching to e-cigarettes (e-cigarettes/vapes/JUULs) and no longer using traditional cigarettes is*… Drawing upon previous research on the reasoned action approach [20], a scale was developed by averaging their responses to (0) *not up to me*…. (100) *up to me* and (0) *not under my control*… (100) *under my control* (*N* = 305, *M* = 90.33, SD = 17.49, *r* = 0.80). A confirmatory factor analysis shows that both items load on one component.

### Analysis plan

We determined that random assignment was successful, in that participants did not differ from one another based on assigned condition in their age (*p* = 0.76), gender identity (*p* = 0.84), race (*p* = 0.23), ethnicity (*p* = 0.68), smoking frequency (*p* = 0.14), and past 30-day e-cigarette use (*p* = 0.38). We conducted univariate analyses with Tukey comparisons on each of the three dependent measures (health risk attitudes, injunctive norms, and perceive behavioral control) with label condition (reduced harm message with smoking cue/reduced harm message without smoking cue/and control no message condition) as the predictor. A sensitivity analysis was conducted post hoc to confirm that the message effect findings were the same with and without these covariates, even as some covariates were significant when included in the model (past 30-day e-cigarette usage for both health risk attitudes (*p* < 0.001) and injunctive norms (*p* < 0.001) and gender identity (*p* = 0.007) and race (*p* = 0.001) for health risk attitudes). Additionally, there were no differences between those who attempted to quit versus those who did not on our outcome measures: health risk attitudes: *F* (1, 303) = 0.29, *p* = 0.59; injunctive norms: *F* (1, 303) = 0.06, *p* = 0.80; *F* (1, 303) = 0.16, *p* = 0.69; perceived behavioral control, *F* (1, 303) = 0.16, *p* = 0.69.

It is important to note that we did not ask participants for their quit or transition intentions. While we tested the proximal determinants of behavioral intentions (health risk attitudes, normative perceptions, and perceived behavioral control), we did not measure their intended plans to make the complete switch to e-cigarettes in this study. We address this further in the future research section.

## Results

### Health risk attitudes

**Fig. 2:**
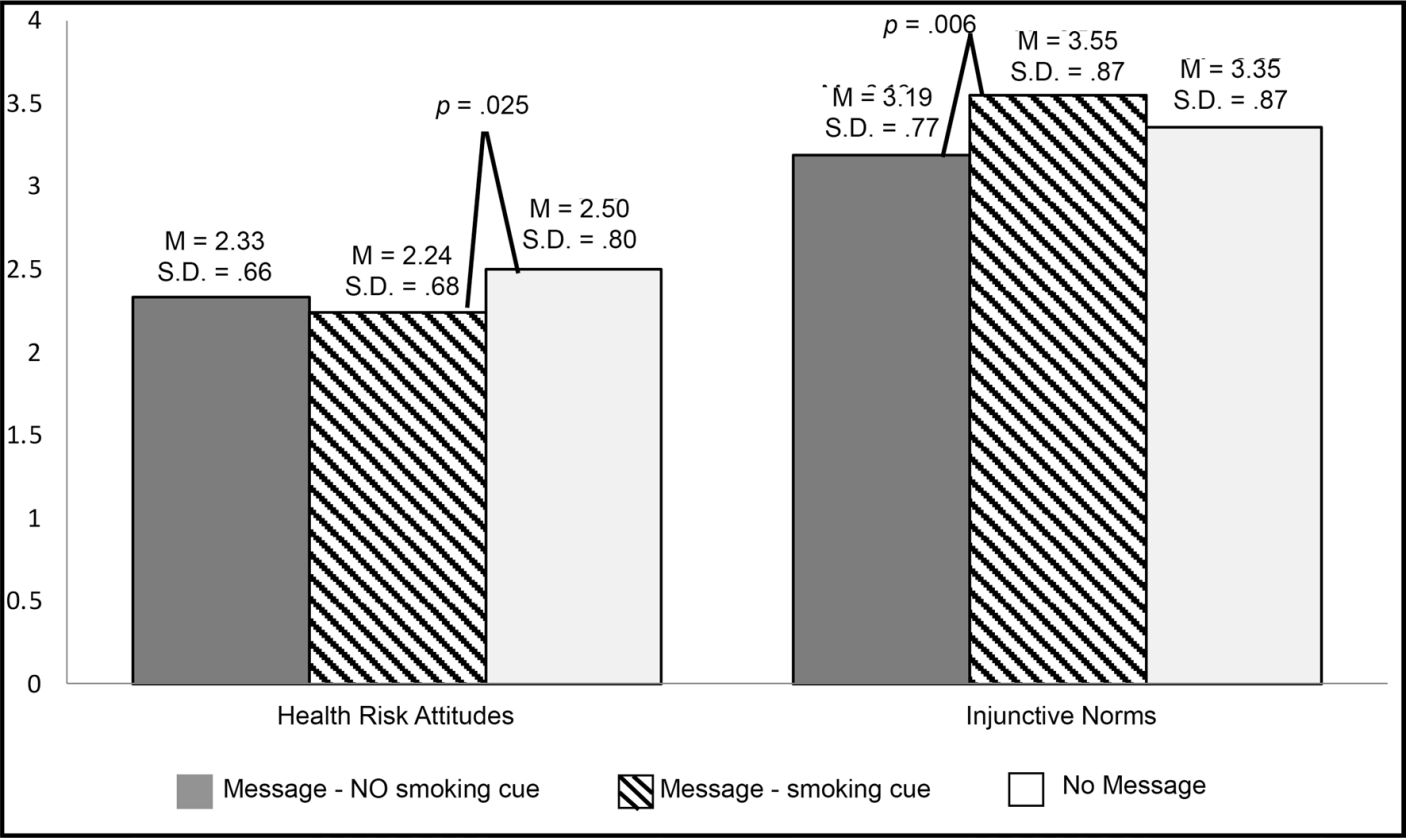
Health risk attitudes and injunctive norms by message condition. *Notes*: Health risk attitudes were measured on the scale (1) not risky at all to (4) very risky. Injunctive norms were measured on the scale (1) strongly disapprove to (5) strongly approve. For presentation purposes, they are including together in one figure, with the vertical axis representing both scales

In support of Hypothesis 1, *F* (2, 302) = 3.50, *p* = 0.03, *η*^2^ = 0.02, participants who viewed the reduced harm message with the smoking cue (*M* = 2.24, SD = 0.68) perceived e-cigarette use as less risky than those who did not see a message (*M* = 2.50, SD = 0.80), *p* = 0.025 (Fig. 2). Participants who viewed the reduced harm message without the smoking cue reported health risk attitudes that were in the middle (*M* = 2.33, SD = 0.66), in response to RQ1. Therefore, informing adults who smoke cigarettes about the reduced toxicant intake associated with making the complete switch from cigarettes to e-cigarettes in a message that includes a smoking cue lowered health risk attitudes.

### Injunctive norms

There was a main effect for label condition on the variable of injunctive norms, *F* (2, 302) = 4.80, *p* = 0.009, *η*^2^ = 0.03. Participants who viewed the reduced harm message with the smoking cue (*M* = 3.55, SD = 0.87) reported higher injunctive norms than those who viewed the message without the smoking cue (*M* = 3.19, SD = 0.77), *p* = 0.006. Participants who did not see the message reported injunctive norms that were in the middle (*M* = 3.35, SD = 0.87) of the other two conditions. Therefore, H2 is only supported for the smoking cue message condition. Upon viewing the reduced harm message with the smoking cue, participants perceived higher beliefs that people they care about would want them to switch from cigarettes to e-cigarettes.

### Perceived behavioral control

There was no difference in perceived behavioral control by message condition, *F* (2, 302) = 0.64, *p* = 0.53. In other words, regardless of whether participants viewed the reduced harm message with or without the smoking cue, or no message at all, they did not differ in the extent to which they said making the complete switch was *up to them* or *under their control*. At first glance, we may be concerned that participants do not feel that they can control whether they make the complete switch, but it turns out that the opposite is true. There is a ceiling effect, with participants scoring very high on average (*M* = 90.33, SD = 17.49) on the 100-point scale.

## Discussion

Participants who viewed the reduced harm message that featured a smoking cue reported the lowest health risk attitudes of e-cigarettes and the highest injunctive norms associated with their use. The messages included both direct claims of lower toxicant intake, as well as the story of a person who had made the complete switch from smoking cigarettes to vaping. While the lower toxicant intake claims likely lowered health risk attitudes, the use of an “individual use depiction” is a message feature that might have increased injunctive norms [18]. As mentioned above, prior research has found that portrayals of an individual in relation to the product are associated with higher injunctive norms [18].

The smoking cues may have connected the participant to either their smoking or vaping behavior, strengthening the message. Therefore, in developing a message that encourages adults who smoke to make the complete switch, it is important to consider using smoking cues. This finding is, in fact, different from some of the recommendations previously in the literature. For example, as noted above, research on smoking, vaping and neutral cues in anti-smoking PSAs and e-cigarette advertisements has been mixed [13–15]. However, the strategic purpose is different for the reduced harm messages in this study, as the message is offering an alternative nicotine product to cigarettes. Therefore, it is likely that these cues work differently here, connecting the message more closely to the experience of smoking, while also connecting to e-cigarette use and the reduced harm message.

When we think about cigarette cessation, often people struggle to be able to quit, even when they want to [21]. However, in the case of making the complete switch from combustible cigarettes to e-cigarettes, participants across all message conditions reported high levels of perceived behavioral control. It appears that participants may be overconfident about their ability to make the complete switch to e-cigarettes. Over half of the participants had not tried to quit smoking cigarettes yet, and so they may not have realized how hard it can be.

### Implications

One implication of this study is that a brief exposure to a message informing adults of the reduced toxicant intake associated with making the complete switch from cigarettes to e-cigarettes facilitated attitudes and perceptions that are consistent with making that switch. The potential for e-cigarettes to play a role in tobacco control relies on adults switching completely as a reduced harm path. This study tested some message strategies, with promising results. However, it is important to test whether people who only smoke cigarettes and do not vape would respond to messages about e-cigarettes as a reduced harm alternative to cigarettes in the same way as those who already both smoke and vape. Recent research on very low nicotine content (VLNC) cigarettes found differences among these groups, as participants who used more than one nicotine product already were more responsive to messages about alternative products [22].

It is important to note that a message cue may be helpful in a particular strategic message context, even when it has not been in others. The findings show that the condition that paired a smoking cue with a reduced harm message yielded the lowest health risk attitudes and the highest injunctive norms. Therefore, message developers might consider using smoking cues in their adult-targeted PSAs about making the complete switch; however, it is also important to determine whether these message elements have unintended effects on youth if they are exposed to the message.

### Limitations

While we made every effort to be rigorous in our study, there are a few limitations to report. First, this study represents a brief exposure to a single message in which the participant was given time to read the message thoroughly. Future research could determine whether this type of message would garner attention and be effective in the natural environment and whether the effect could be replicated with similar messages, due to the limitations of single message tests [23]. Another limitation of this study is that the sponsor of the message used in the stimuli is unclear, and prior research has found that adults in the USA trust public health sources for information about e-cigarettes, rather than companies associated with the commerce of these products [24]. Therefore, researchers could test whether attributing the message to a trusted public health source could strengthen the findings.

While the effect sizes, *η*^2^ = 0.02 and 0.03, found in this study might seem low, this is in line with the effect sizes found in similar studies featuring a brief message exposure [25]. Health communication makes the general assumption that the effect of this brief exposure would be much larger if considered in relation to the many messages individuals would be exposed to over the course of a public health campaign or within their daily lives. Furthermore, a meta-analysis on the effect of public health campaigns has found that effect sizes tend to be lower for addictive behaviors, and particularly for smoking cessation campaigns [26].

Another limitation of this study is that we only looked at how adults who smoke cigarettes and adults who smoke cigarettes and vape perceived the message. Future research could consider how youth would perceive this message should they be exposed to it. For example, would the toxicant indicator message make youth think that the product is safe for them, and would the smoking cue make them more interested in e-cigarettes?

As noted above, we selected to focus on injunctive norms, rather than descriptive norms, as injunctive norms might be particularly influential for messages about cigarette cessation since there is a “threat of social disapproval for inappropriate behavior” [27, p. 264]. Descriptive norms, on the other hand, might be more useful for new behaviors, and e-cigarette use at the time of this study was not a new behavior, particularly among adults who smoke cigarettes [18, 27]. That said, future research could test whether this message would influence descriptive norms as well, particularly as prior research has found an influence of descriptive norms on smoking behavior [28].

As mentioned above, this study did not actually measure behavioral intentions or behavior, as a choice was made early on to only measure the influence of the message on the three concepts we investigated. Prior research has established that the proximal variables measured are good indicators of behavioral intentions, with meta-analytic work demonstrating the importance of attitudes in predicting intentions in health contexts like condom use, as well as smoking intentions and with specific research on vaping demonstrating the importance of attitudes and perceived norms, using a scale that included injunctive norms, in predicting behavioral intentions, which in turn predicted behavior in this context [29–31]. Future research could determine which of the proximal determinants (attitudes, perceived norms, and perceived behavioral control) are most important in predicting intentions and informing health message design in this specific context, and the findings in this study offer results that are indicators of intent and can inform this future research. Finally, the literature on the reasoned action approach and the integrated behavioral model includes additional concepts, such as the salience of the behavior and environmental constraints, and future research could investigate these variables [16].

E-cigarette use has been shown in previous research to support cigarette cessation [7], although studies have shown that people who smoke are sometimes concerned about the health risks of e-cigarettes and whether they can help with cigarette cessation [3–5]. In this study of adults, we demonstrated that a reduced toxicant intake message with a smoking cue that portrays an individual making the complete switch from cigarettes to e-cigarettes facilitated lower e-cigarette health risk attitudes and higher injunctive norms. This study suggests that reduced harm messages about e-cigarettes could support adults who smoke cigarettes in making the complete switch to e-cigarettes.

## Data Availability

We have submitted a data file to OSF. The link to the public data set is http://osf.io/VB4KD.
